# Supplementary far-red light enhances the quality and root development of double-root-cutting grafted watermelon seedlings

**DOI:** 10.3389/fpls.2025.1586698

**Published:** 2025-09-03

**Authors:** Cuinan Wu, Yaya Wang, Kai Cao, Encai Bao, Haiyan Ding, Xiaoting Zhou, Yifan Dong, Shipu Xu, Xue Wu

**Affiliations:** ^1^ The Agriculture Ministry Key Laboratory of Agricultural Engineering in the Middle and Lower Reaches of Yangtze River, Institute of Agricultural Facilities and Equipment, Jiangsu Academy of Agricultural Sciences, Nanjing, Jiangsu, China; ^2^ College of Horticulture, Xinjiang Agricultural University, Urumqi, Xinjiang, China; ^3^ School of Public Health, Dali University, Dali, Yunnan, China; ^4^ College of Horticulture, Sichuan Agricultural University, Chengdu, Sichuan, China; ^5^ School of Biosciences, University of Nottingham, Nottingham, United Kingdom; ^6^ Key Laboratory of Smart Agricultural Technology (Yangtze River Delta), Ministry of Agriculture and Rural Affairs, Shanghai, China

**Keywords:** watermelon, double-root-cutting grafting, root regeneration, far-red light, seedling growth

## Abstract

**Introduction:**

The regeneration of roots is crucial for the survival and healthy development of double-root-cutting (DRC) grafted watermelon seedlings. However, methods to effectively enhance root regeneration in the rootstock of DRC grafted watermelons remain unclear.

**Methods:**

In this study, supplementary far-red (FR) light was applied to DRC grafted watermelon seedlings to evaluate its impact on seedling growth and rooting, using dark (CK) as the control.

**Results and discussions:**

It was discovered that supplementary FR light substantially promoted root development in the rootstock, reducing the time required for root regeneration and boosting root biomass. Transcriptome profiling indicated that genes associated with sugar catabolism, oxidative stress, and auxin signaling were markedly upregulated in roots by FR light at 4 d post-grafting. FR_0.3_ (red/far-red ratio = 0.3) light significantly enhanced the expression of genes involved in hyperoxide scavenging (*CmAPX1*, *CmPOD1*, *CmCAT1*), sugar transportation (*CmSWEET12*, *CmBST2*, *CmSCP1*), and auxin response (*CmAUX28, CmIAA11*, *CmSAUR20*) compared with the control. Moreover, FR_0.3_ light treatment notably decreased reactive oxygen species content and improved antioxidant enzyme activities in roots compared with the control. However, despite increased gene expression, peroxidase and catalase did not contribute substantially to reactive oxygen species scavenging at the protein activity level under FR_0.3_ compared with other light qualities. In addition, sugar content and hexokinase activity responded differently to light quality: starch, sucrose, and hexokinase activity were significantly increased by FR_0.3_ light at 4 d post-grafting, while glucose content in the FR_0.3_ treatment was significantly higher than that in other treatments only at 8 d post-grafting. These results demonstrate that supplementary FR light significantly promotes rooting and growth of DRC grafted watermelon seedlings. Specifically, FR light can induce root regeneration in the rootstock, potentially by alleviating oxidative stress during grafting and providing a relatively stable plant environment through the synergistic effects of sugar metabolism, the antioxidant enzyme system, and auxin accumulation in the roots via the regulation of antioxidants, sugar metabolism, and auxin-related gene transcription. The findings from this study present a practical method to enhance the quality of grafted watermelon seedlings.

## 1 Introduction

Watermelon (*Citrullus lanatus*) is intensively cultivated worldwide as an economically important horticultural crop (Li H. et al., 2021; Ulas, 2022). China is the world’s largest watermelon producer (63.0 million tons), followed by Iran (4.1 million tons) and Turkey (4.0 million tons), with a combined contribution of 70.4% of the world’s watermelon production (101 million tons) (Food and Agriculture Organization, 2024). Grafting has become a routine technique in watermelon production to enhance fruit yield, fruit quality, and resistance to biotic and abiotic stresses (Mashilo et al., 2023; Yavuz et al., 2020; Nawaz et al., 2018; Lu et al., 2022). Compared with conventional grafting, double-root-cutting (DRC) grafting, which involves cutting off the roots of both the scion and rootstock, has the advantages of faster grafting speed, higher survival rate, and better rootstock rooting (Wu et al., 2024; Sun et al., 2020). Root regeneration is the key factor determining the survival of DRC grafted seedlings (Sallaku et al., 2022; Schwarz et al., 2013; Böhm et al., 2017). Therefore, understanding how to effectively promote root formation and increase survival rate is of critical importance.

Apart from temperature and relative humidity, which are well-recognized environmental factors, light has been proposed as a significant factor influencing the survival and growth of grafted seedlings. Precise control of light wavelengths offers the potential to fine-tune morphological and physiological responses and enhance seedling quality in grafted plants. Currently, several studies have examined the effect of light quality on the growth of grafted seedlings (Yousef et al., 2021; Vu et al., 2014; Yang et al., 2013). For instance, red light (R) was beneficial for vascular reconnection in grafted tomato seedlings (Yousef et al., 2021; Vu et al., 2014), whereas a mixture of blue and red light was essential for the healing and acclimatization of pepper (Kwack et al., 2021; Yang et al., 2013). Moreover, the addition of 30% green light to red and blue light promoted graft reunion, rootstock rooting, and growth of DRC grafted tomato seedlings (Li F.H. et al., 2021). In grafted watermelon seedlings, blue light was shown to promote healing through improved photosynthetic performance (Moosavi-Nezhad et al., 2021). Bantis et al. (2021) reported that adding 12% blue light to red light expedited graft healing and boosted the physiological response and quality of grafted watermelon seedlings. Recently, there has been increasing interest in examining the impact of incorporating far-red (FR) light on the growth, quality, and stress tolerance of vegetable crops. By modulating FR light levels (R/FR ratio), the interconversion between the red light–absorbing (Pr) and far-red light–absorbing (Pfr) forms of phytochromes can be triggered, influencing plant morphogenesis and physiological responses (Kim et al., 2023; Meijer et al., 2023). Supplementary FR light treatment has been reported to improve root development and vegetative growth of grafted watermelon and tomato seedlings (Bantis et al., 2022b; Wei et al., 2018). FR light significantly upregulated the expression of *CmGH9B14* (a member of the *GH9B* family, encoding β-1, 4-glucanase and functioning as a key gene involved in cell wall remodeling and intercellular adhesion in grafted watermelon seedlings) (Zhu et al., 2023), induced the expression of phytochrome-interacting factor genes (*CmPIFs*, which interact with phytochromes and other regulatory proteins to adjust the expression of genes necessary for root formation and development), and activated the transcription of auxin-related genes (Wu et al., 2024), leading to activation of the auxin pathway and promotion of healing and root regeneration in pumpkin-grafted watermelon seedlings (Wang et al., 2024). However, research into the impacts of FR light on root regeneration in DRC grafted watermelon seedlings is limited, and the mechanism by which FR regulates their growth remains unknown.

Root regeneration in grafted seedlings is a complex process involving regulation of metabolism and hormone signaling (Zhang et al., 2023). Grafting may induce reactive oxygen species (ROS) overproduction and cause lipid peroxidation (Wang et al., 2007). Antioxidant enzymes such as peroxidase (POD), superoxide dismutase (SOD), catalase (CAT), and ascorbate peroxidase (APX) can efficiently scavenge the harmful effects of ROS, thereby preventing membrane system peroxidation and cellular damage (Wang et al., 2024). The signaling cascade between phytohormones such as auxin and others has also been implicated in blue light–promoted healing and root development in grafted watermelon (Bantis et al., 2021). Christiaens et al. (2019) revealed that FR could stimulate adventitious root formation of *Chrysanthemum* by increasing auxin transport. Wu et al. (2024) reported that FR induced adventitious root formation in rootstocks of grafted watermelon seedlings, potentially through auxin accumulation. In addition, an adequate supply of carbon is required for root formation (Klopoteka et al., 2012). Studies on root formation in *Petunia* × *hybrida* cuttings revealed the involvement of carbohydrates in the early stage of root formation, with increased sucrose translocation from source leaves to the stem base (Ahkami et al., 2009). In this respect, carbohydrate status is expected to play a major role in the healing and rooting of grafted seedlings (Moosavi-Nezhad et al., 2021). Notably, the ability of rootstock to regenerate roots directly affects the quality of grafted seedlings. However, according to the available literature, the mechanism of root regeneration of rootstock in vegetable crops remains unclear. Moreover, interactions among FR, ROS removal, sugar metabolism, plant hormones, and root formation in rootstocks have not been completely determined.

Our recent experience suggests that FR light benefits root regeneration in DRC grafted watermelon (Wu et al., 2024; Wang et al., 2024). In the present work, we examined the role of FR-mediated synergistic effects of ROS, sugars, and auxin in the rooting process of DRC grafted watermelon. Differentially expressed genes in response to light treatments were identified, and the expression of genes related to oxidative stress, and sugar and auxin metabolism during root regeneration was analyzed. The results suggest that these genes may synergistically mitigate grafting stress, aid in sugar transport to the roots for energy and carbon skeletons and activate auxin-related pathways under FR treatment. We also investigated ROS and sugar contents, and activities of antioxidant enzymes and enzymes associated with sugar transport and metabolism in the roots. The findings characterize the relationship between light conditions and root formation in DRC grafted watermelon seedlings, offering potential guidelines for enhancing seedling cultivation practices.

## 2 Materials and methods

### 2.1 Plant material, grafting, and light treatment

#### 2.1.1 Plant growth and grafting

The commercial watermelon (*Citrullus lanatus*, Zaojia 8424) was used as the scion, and pumpkin (*Cucurbita moschata*, Kangbingxianfeng) was used as the rootstock. The seeds of the scion and rootstock with uniform size were selected, disinfected, and soaked in a dark incubator at 25°C for 3–5 h. Seeds were then sown in a plastic plug tray with 72 cells (54 × 28 cm). Watermelon seeds (scion) were sown 2 d after the pumpkin seeds (rootstock) to allow the seedlings to develop similar stem diameters for the graft union. The seedlings were grown in a controlled environment chamber at 26/18°C day/night, light intensity of 200 μmol·m^-2^·s^-1^, and relative humidity of 50%–70%. The photoperiod was set to 12/12 h day/night.

When the cotyledon of the scion was fully expanded and the rootstock had one true leaf (14-day-old rootstock seedlings and 12-day-old watermelon seedlings), DRC grafting was performed (**Supplementary Figure S1**). The rootstock, with an average plant height of 13.02 ± 0.39 cm and average stem diameter of 2.40 ± 0.13 mm (n=30), was cut 4–6 cm below the cotyledon. Half of each rootstock cotyledon was removed to avoid mutual shading by neighboring seedlings. A ‘V’ shaped incision was cut on the scion 1–2 cm below the cotyledon segment. A grafting needle was then used to create a hole approximately 0.5–1.0 cm deep in the incision on the rootstock, into which the scion was carefully inserted and held in place with a plastic grafting clip (Li H. et al., 2021; Wu et al., 2024; Wang et al., 2024). The grafted seedlings were planted in a 72-cell plastic plug tray (54 × 28 cm) and immediately covered with a matching transparent tray lid to maintain humidity. The seedling substrate (Huai’an Chaimihe Agricultural Science and Technology Co. LTD) was used for plant growth. The electrical conductivity and pH of the substrate were 3.6 mS/cm and 6.3, respectively, with an organic content of 22.1% and total nutrient content (N+P_2_O_5_+K_2_O) of 3.5%. Following standard production practice, the relative humidity was maintained at over 90% for the first 3 d post-grafting to prevent leaf dehydration, gradually reduced to 80% at 4–6 d post-grafting and 70% at 7–10 d post-grafting, after which the covering lid was removed.

#### 2.1.2 Light treatment

Different light treatments were conducted from the first day after grafting until 10 d after grafting. In total, four light quality treatments were established, with dark (CK) as the control and additional FR light as treatments (**Table 1**). FR was added to white light, whose R/FR ratio was 6.4 (FR_6.4_), so that the R/FR ratio decreased to 3 (FR_3.0_) or 0.3 (FR_0.3_). The control referred to the dark treatment during the first 3 d after grafting.

**Table 1:** Light intensity (μmol·m^-2^·s^-1^) used in different light treatments during growth stages of 0 - 3, 4 - 6, and 7–10 days after grafting.

Light treatments	Light intensity/(μmol·m^-2^·s^-1^)		
	0–3 d	4–6 d	7–10 d
CK	0	100	200
FR_6.4_	50	100	200
FR_3.0_	50	100	200
FR_0.3_	50	100	200

CK refers to the dark treatment during the first 3 d after grafting. FR_6.4_ represents white light with a red to far-red (R/FR) ratio of 6.4. FR_3.0_ and FR_0.3_ represents treatment in which far-red was added to white light to decrease the ratio of red/far-red light to 3.0 and 0.3, respectively.

**Figure 1:**
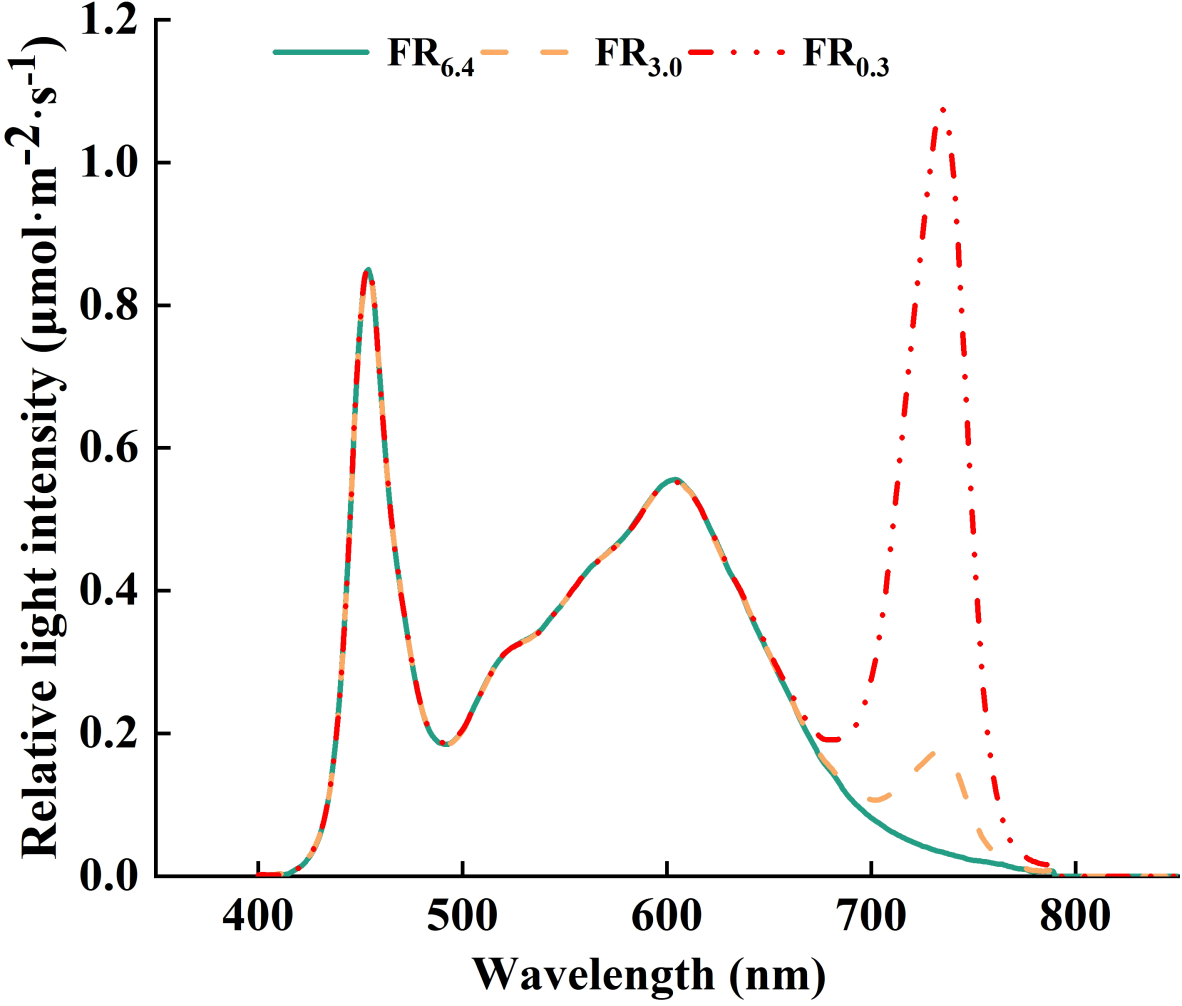
Spectral distribution characteristics of light treatments. FR_6.4_ represents white light with a red to far-red ratio of 6.4. FR_3.0_ and FR_0.3_ represents for adding far-red light to the white light to decrease the red/far-red ratio to 3.0 and 0.3, respectively.

All supplementary lighting was provided by LED top light modules (Nanjing Hengyu Instrument and Equipment Co., Ltd., China). The light intensity and spectrum of all light treatments were measured using a spectroradiometer (PS-300, Apogee Instruments Inc., Logan, UT, USA) (**Figure 1**). Light intensity was gradually increased to allow the grafted seedlings to acclimate to the environment (Li F.H. et al., 2021; Sun et al., 2020; Bantis et al., 2020). In the first 3 d after grafting, plants were treated with low light (50 μmol·m^-2^·s^-1^, except for CK). During 4–6 and 7–10 d after grafting, the light intensities were increased to 100 and 200 μmol·m^-2^·s^-1^, respectively. From day 11 onwards, the seedlings were subjected to standard management in a growth chamber with white light of 200 μmol·m^-2^·s^-1^ and watered every 3 d with Hoagland nutrient solution.

The experiment followed a completely randomized design, with each treatment utilizing 36 seedlings per replication, and all treatments were replicated three times.

### 2.2 Calculation of survival rates and growth index

The survival rate of grafted seedlings under different light treatments was evaluated at 10 d after grafting using the following formula:


$$\text{Survival rate }(\%)=\frac{\text{Number of survived grafted seedlings}}{\text{Total number of grafted seedlings}}\times 100$$

The growth index of grafted watermelon seedlings was analyzed at 8 and 20 d after grafting. Fresh and dry weights were measured using electronic scales (0.0001 g). The heights and diameters of the scion and rootstock were measured with a ruler (1 mm) and vernier caliper (0.02 mm), respectively. The root-to-shoot ratio was calculated as the ratio of root fresh weight to shoot fresh weight at 20 d after grafting. A total of 10 seedlings were used in each treatment for measurement at each time point, and all treatments were replicated three times. The above measurements were also used to calculate the seedling index at 20 d after grafting, which was calculated as follows:


$$\text{Seedling index}=(\frac{\text{stem diameter }(\text{mm})}{\text{seedling height }(\text{mm})}+\frac{\text{root dry weight }(\text{g})}{\text{shoot dry weight }(\text{g})})*\text{whole plant dry weight }(\text{g})$$

### 2.3 Root vigor

Root vigor was measured using the triphenyl tetrazolium chloride (TTC) method at 4, 8, 12 and 20 d after grafting (Zhang et al., 2013). At each time point, three DRC grafted seedlings were sampled for each treatment, with three replications. Root vigor was calculated as the ratio of the TTC reduction amount (μg) to fresh root weight (g) over a certain time (h).

### 2.4 Measurements of ROS, antioxidant enzyme activities, and sugar content

Root physiological parameters, including ROS levels, antioxidant enzyme activities, and soluble sugar/starch content, were analyzed at 0, 4, 8, 12, and 20 d after the initiation of light treatments. At each time point, three DRC grafted seedlings were sampled for each treatment, with three replications. Since adventitious roots were absent in both the CK and FR_6.4_ treatments at 4 d after grafting, root tissues were sampled from the 1.5 cm zone surrounding the incision site—a region primed for adventitious root emergence (Zhang et al., 2023; Sun et al., 2020; Li F.H. et al., 2021).

Fresh root samples (0.2 g) were homogenized in 5 mL of ice-cold 50 mM phosphate buffer (pH 7.0) and centrifuged at 15,000 × g for 15 min at 4 °C. The resulting supernatant was collected for subsequent analyses. SOD activity was assayed via nitro blue tetrazolium (NBT) photochemical reduction inhibition (Wang et al., 2021; Li F.H. et al., 2021). CAT and POD activities were quantified by monitoring hydrogen peroxide (H_2_O_2_) hydrolysis at 240 nm and guaiacol oxidation at 470 nm, respectively (Cakmak and Marschner, 1992). APX activity was determined based on ascorbate oxidation kinetics at 290 nm (Nakano and Asada, 1981; Bai et al., 2023).

Soluble sugars and starch were extracted and measured using UV spectrophotometry (UV-2800A, UNICO, Shanghai, China) following Shen et al. (2019) and Bai et al. (2023). Glucose, sucrose, hexokinase (HXK), sucrose synthase (SuSy), H_2_O_2_ and superoxide anion (O_2_^-^) levels were determined using commercial assay kits (Solebo Biotechnology Co., Ltd.) according to the manufacturer’s protocols. All assays included three technical replicates per biological sample.

### 2.5 RNA extraction, transcriptome sequencing, and quantitative RT-PCR validation

Based on preliminary data indicating maximal light-induced transcriptional responses at 4 d post-treatment (Wu et al., 2024), shoot and root tissues under CK (dark), FR_6.4_ (white light), and FR_0.3_ (far-red light) conditions were sampled for transcriptome profiling, with three seedlings per treatment and three replications. Total RNA was isolated using the FastPure Universal Plant Total RNA Isolation Kit, and RNA integrity was verified by spectrophotometry (A_260_/A_280_ nm > 1.9).

RNA-seq libraries were prepared from DNase-treated RNA (5 μg per sample) and sequenced on an Illumina platform (Biomics Biotechnology Co., Ltd., China). RNA samples with high purity were used to construct cDNA libraries, and their quality and quantity were verified using an Agilent 2100 Bioanalyzer. The qualified cDNA libraries were sequenced using the Illumina platform (Biomics Biotechnology Co., Ltd., China). Raw reads in FASTQ format were first processed to obtain high-quality clean reads, which were then *de novo* assembled into gene sets separately. Gene expression levels were quantified using FPKM (fragments per kilobase of transcript per million mapped fragments) values. Genes were then clustered based on their expression patterns using the K-means method. Differentially expressed genes (DEGs) among samples were identified using DESeq2. To determine putative biological functions and pathways for the DEGs, Gene Ontology (GO) and Kyoto Encyclopedia of Genes and Genomes (KEGG) enrichment analyses were performed. GO terms and KEGG pathways with q-values < 0.05 were considered significantly enriched.

For qRT-PCR validation, cDNA was synthesized from 4 d treated samples (CK, FR_6.4_, FR_0.3_) using reverse transcriptase (Thermo Scientific, Lithuania). Primers for target genes (**Supplementary Table S1**) were designed with Primer 6.0, with *CmActin* as the reference gene. Reactions (20 μL total volume) contained 2 μL cDNA, 0.4 μL each of forward/reverse primer (10 μM), 10 μL 2× ChamQ Universal SYBR qPCR Master Mix, and 7.2 μL nuclease-free H_2_O. Amplification was performed on a Light Cycler 96 system (Roche, Switzerland) following the cycling conditions described by Wu et al. (2024). Relative gene expression was calculated using the 2^-ΔΔCt^ method (Livak and Schmittgen, 2001), with three biological and technical replicates per treatment.

### 2.6 Statistical analysis

All experimental data were processed and analyzed using SPSS 26.0 (IBM, Armonk, NY, USA) and Origin 2021 (Northampton, MA, USA). One-way analysis of variance (ANOVA) with Duncan’s multiple range test at *p* < 0.05 was used to detect differences among treatment groups on the same day.

## 3 Results

### 3.1 Rooting, survival, and growth of DRC grafted watermelon seedlings

**Figure 2:**
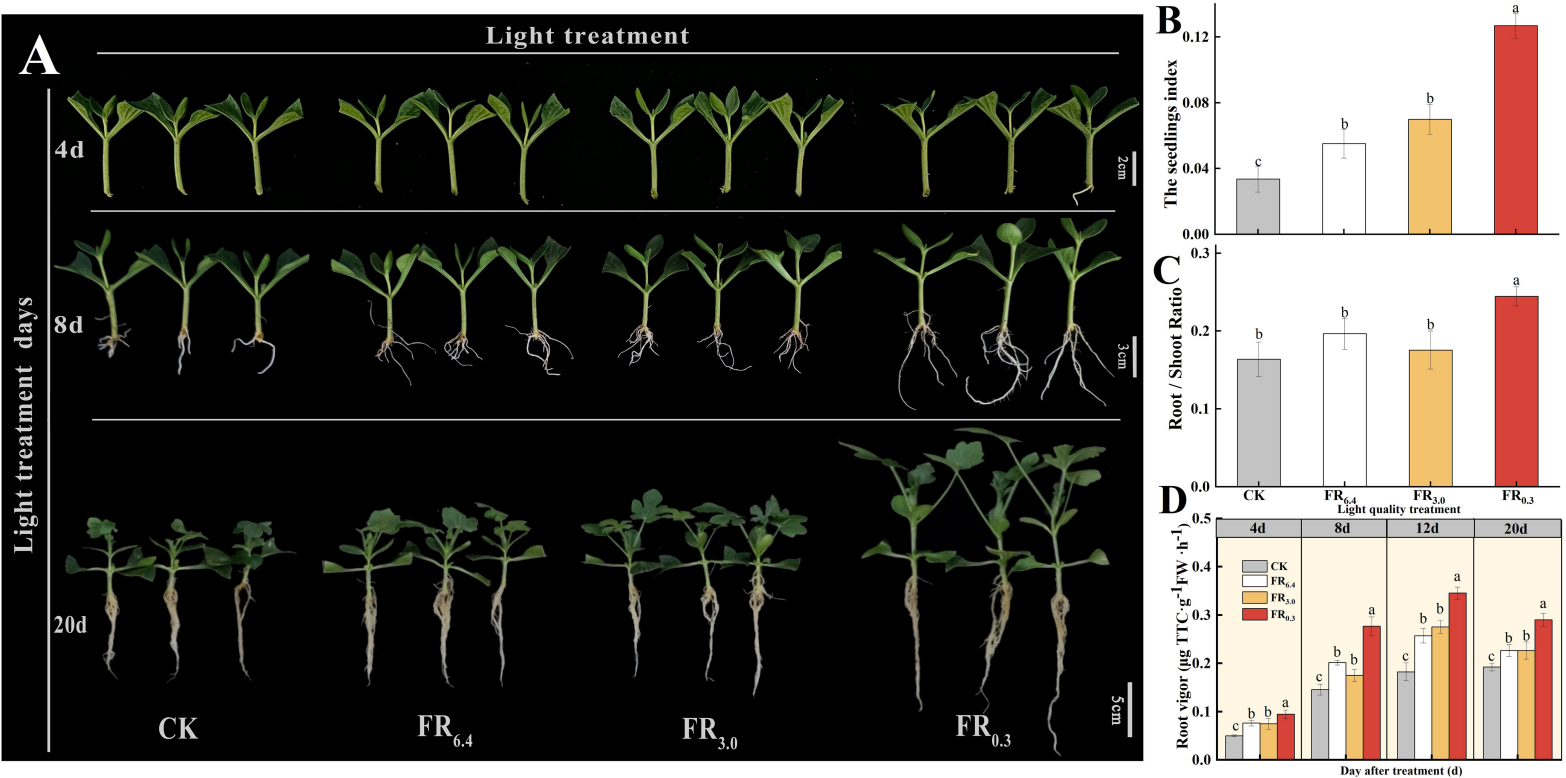
Growth and phenotype of double-root-cutting (DRC) grafted watermelon seedlings under different light quality treatments. **(A)** Phenotype at 4, 8, and 20 days after grafting. Vertical axis: days after treatment; horizontal axis: light treatments. Scale bar = 2 cm for 4 d, 3 cm for 8 d, 5 cm for 20 d. **(B–D)** show seedling index, root/shoot ratio, and root vigor, respectively. Data for seedling index **(B)** and root/shoot ratio **(C)** are means ± standard deviation (SD) from three independent experiments with 10 biological replicates each (n = 30). Data for root vigor **(D)** are means ± SD from three independent experiments with three biological replicates each (n = 9). Different letters indicate significant differences at *P* < 0.05 according to Duncan’s multiple range test and the significant differences were compared among light treatments at indicated time point. CK: dark control; FR_6.4_: white light; FR_3.0_: R/FR = 3.0; FR_0.3_: R/FR = 0.3.

As shown in **Figure 2**, root regeneration of DRC grafted watermelon seedlings was accelerated by supplementary FR light. FR_0.3_-treated seedlings began to develop roots at 4 d after grafting, whereas plants under other light treatments exhibited only minimal root emergence (**Figure 2A**). DRC grafted seedlings had the longest roots and highest root weight after 8 d under FR_0.3_ treatment (**Figure 2A**). As seedlings grew, the effect of FR light on plant growth became more pronounced. Root dry weight, scion dry weight, and rootstock dry weight per plant were significantly higher under FR_0.3_ compared with seedlings exposed to other light treatments at 20 d after grafting (**Supplementary Table S2**).

The seedling index and root/shoot ratio were further investigated at 20 d after grafting to verify the function of FR light on root generation (**Figures 2B, C**). Compared with CK, FR0.3 treatment significantly increased the seedling index (**Figure 2B**) and root/shoot ratio (**Figure 2C**) by 278% and 49% respectively. Besides, seedlings treated with FR0.3 showed the highest root vigor during the measurement period (**Figure 2D**), which further proved the positive effect of FR on grafted watermelon seedlings.

**Figure 3:**
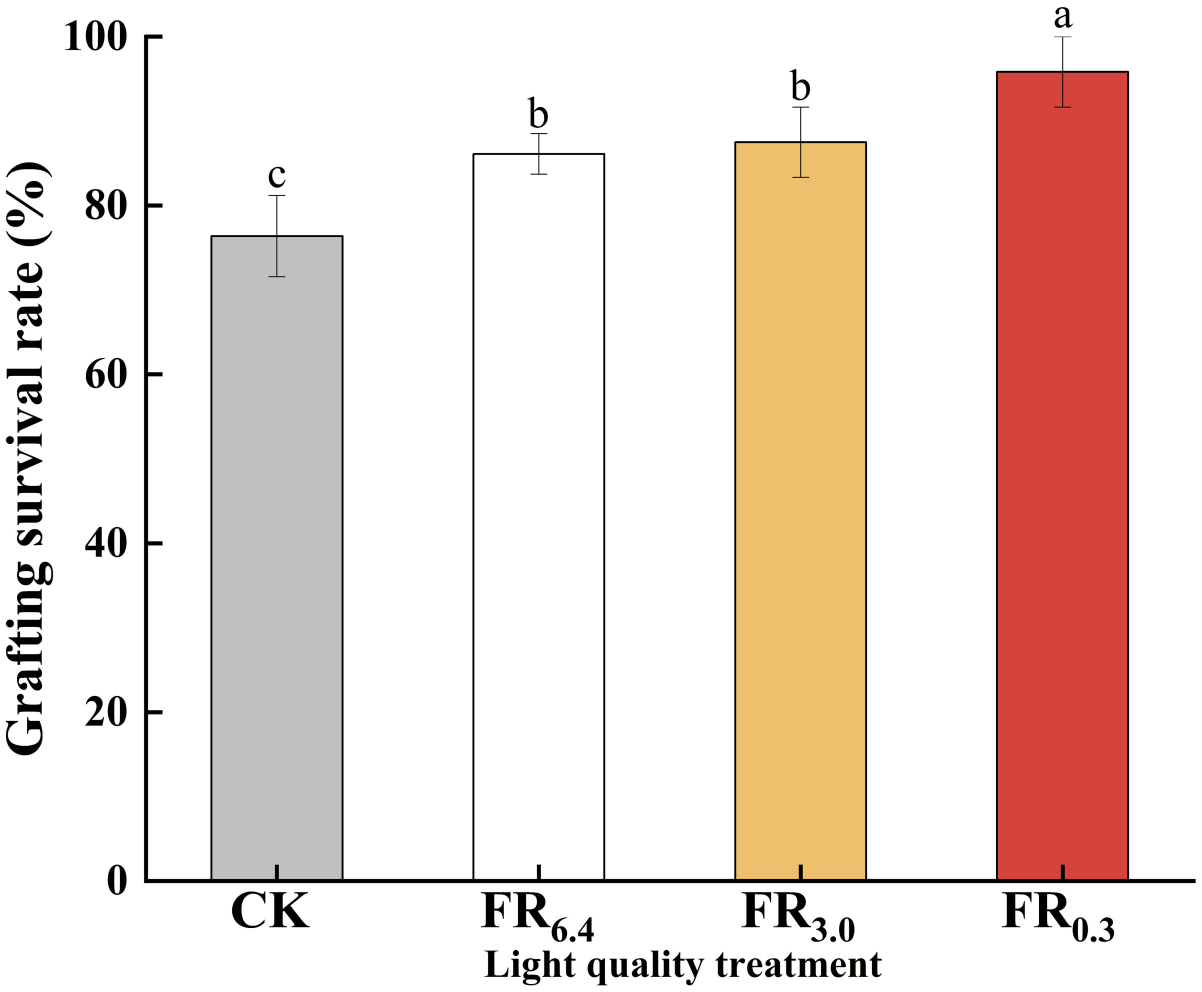
Survival rate of double-root-cutting (DRC) grafted watermelon seedlings under different light quality treatments. Statistical significance was determined by Duncan’s test (*P* < 0.05). Data are shown as means ± standard deviation (SD) from three independent experiments with 36 biological replicates each (n = 3). Different letters indicate significant differences at *P* < 0.05 according to Duncan’s multiple range test and the significant differences were compared among light treatments. CK: dark control; FR_6.4_: white light; FR_3.0_: R/FR = 3.0; FR_0.3_: R/FR = 0.3.

Analysis of survival rates revealed that survival was significantly higher under light conditions (over 86%) than under dark conditions (CK, 76.4%) (**Figure 3**). Among the treatments, FR_0.3_ achieved the highest survival rate (95.8%), followed by FR_3.0_ (87.5%) and FR_6.4_ (86.1%). No significant differences were observed in survival rate or growth parameters between the FR_3.0_ and FR_6.4_ treatments.

**Figure 4:**
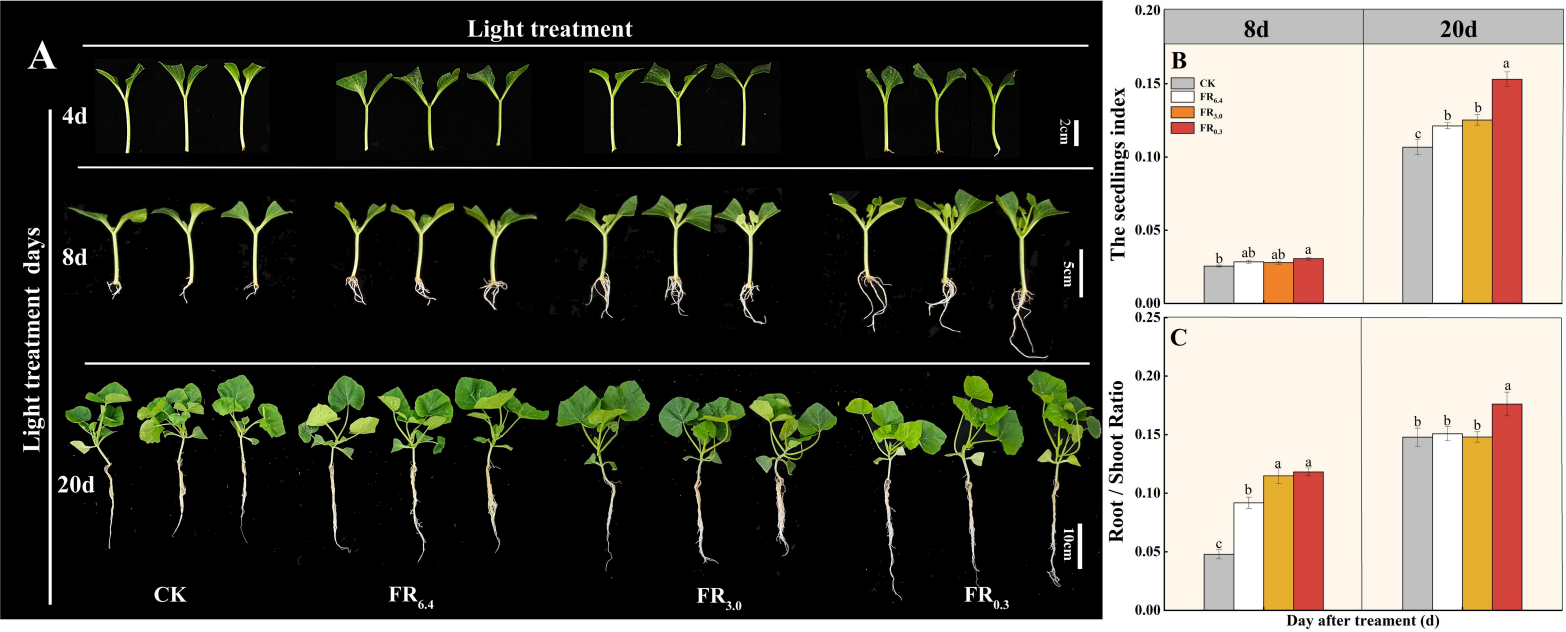
Growth and phenotype of excised rootstock (pumpkin) seedlings under different light quality treatments. **(A)** showed the seedling phenotype for 4, 8 and 20 d after cutting. Vertical axis: days after treatment, Horizontal axis: light treatments. Scale bar = 2 cm for 4 d, Scale bar = 5 cm for 8 d, Scale bar = 10 cm for 20 d. **(B, C)** represented the seedling index and root/shoot ratio, respectively. Data are shown as means ± standard deviation (SD) from three independent experiments with 10 biological replicates each (n = 30). Different letters indicate significant differences at *P* < 0.05 according to Duncan’s multiple range test and the significant differences were compared among light treatments at each time point. CK: dark control; FR_6.4_: white light; FR_3.0_: R/FR = 3.0; FR_0.3_: R/FR = 0.3.

Further, rootstock (pumpkin) seedlings were used to verify the effect of FR on root formation. Excised pumpkin seedlings (prepared following the same procedure as DRC grafting but without the watermelon scion) were subjected to different R/FR ratio treatments. The results were consistent with those for DRC grafted watermelon seedlings. Supplementary FR light (FR_0.3_) expedited root development in pumpkin seedlings compared with other light treatments at 4 d after grafting (**Figure 4A**). In addition, FR_0.3_ treatment boosted both aboveground and belowground growth of excised pumpkin seedlings, with significantly increased seedling index, root/shoot ratio, root length, fresh/dry weight, and plant height compared with other light treatments, especially at 20 d post-grafting (**Figures 4B, C**; **Supplementary Table S3**). Notably, at 8 d post-grafting, FR_0.3_ resulted in significantly higher fresh/dry weight of the aboveground part in excised pumpkin seedlings, whereas no significant difference was observed between FR_0.3_ and other light treatments in the fresh/dry weight of the scion in DRC grafted watermelon seedlings (**Supplementary Table S3**).

### 3.2 Transcriptome analyses identified key genes and processes involved in FR light-regulated root development

**Figure 5:**
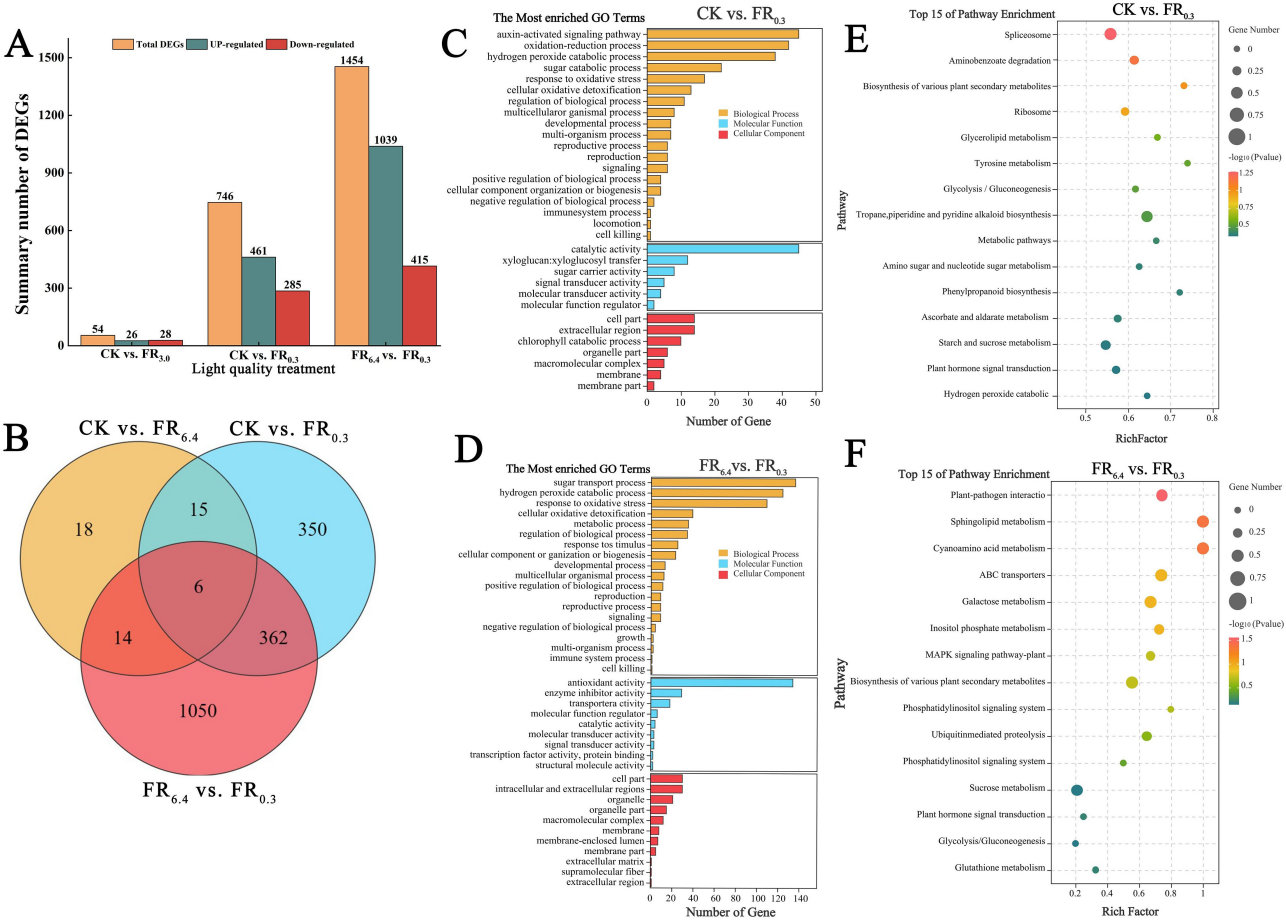
Gene ontology (GO) enrichment and Kyoto Encyclopedia of Genes and Genomes (KEGG) enrichment analyses of differentially expressed genes (DEGs) in the roots of DRC grafted watermelon seedlings under different light quality treatments for 4 d.**(A, B)** Number of DEGs in CK vs. FR_6.4_, CK vs. FR_0.3_, and FR_6.4_ vs. FR_0.3_ comparisons. **(C, D)** Comparative GO terms for CK vs. FR_0.3_ and FR_6.4_ vs. FR_0.3_, respectively. Y-axis: GO term; x-axis: number of DEGs. **(E, F)** KEGG pathways in CK vs. FR_0.3_ and FR_6.4_ vs. FR_0.3_ comparisons, respectively. Y-axis represented pathways, while x-axis represented rich factor. The rich factor meant the degree of DEGs enrichment in each pathway, the bigger the rich factor the greater the DEGs enrichment. The q value was the p value which was corrected for multiple hypothesis testing. The closer to zero the q value was, the more DEGs clustered (0 < q value < 1). The round dots of various sizes represented the number of DEGs clustered in each pathway, the larger the dot, the more the DEGs (*P* < 0.05). The various colors of the dots correspond to the different q value range. CK: dark control; FR_6.4_: white light; FR_0.3_: R/FR =0.3.

RNA-seq was performed to further analyze the molecular mechanisms by which supplementary FR light induced root formation in the rootstock at 4 d after grafting. To identify key genes in this study, three comparison groups were constructed: CK vs. FR_6.4_, CK vs. FR_0.3_, and FR_6.4_ vs. FR_0.3_. Genes showing more than a twofold change in the comparison groups were used in bioinformatics analyses. A total of 1,454 differentially expressed genes (DEGs) were detected in the roots of the FR_6.4_ vs. FR_0.3_ comparison, with 1,039 DEGs upregulated and 415 DEGs downregulated (**Figures 5A, B**). The roots in the CK vs. FR_0.3_ comparison had fewer DEGs, totaling 746, with 461 upregulated and 285 downregulated. Meanwhile, only 54 DEGs were identified in the CK vs. FR_6.4_ comparison. In conclusion, supplementary FR light had a greater influence on the transcriptional levels of root genes than other treatments.

The DEGs were further annotated using Gene Ontology (GO) categories. Three functional components were included in the GO analysis: biological process, molecular function, and cellular component (**Figures 5C, D**). DEGs involved in sugar transport, oxidative stress, and the hydrogen peroxide catabolic process were among the top five GO terms in the biological process and molecular function categories across the three comparison groups. In the CK vs. FR_0.3_ comparison, DEGs associated with auxin-activated signaling, oxidation–reduction, hydrogen peroxide catabolic process, and sugar catabolic process were primarily clustered in the biological process category. Genes associated with catalytic activity, xyloglucan:xyloglucosyl transfer, and sugar carrier activity were clustered in the molecular function category, whereas DEGs involved in cell part, extracellular region, and chlorophyll catabolic process were clustered in the cellular component category (**Figure 5C**). In the FR_6.4_ vs. FR_0.3_ comparison, DEGs associated with sugar transport, hydrogen peroxide catabolic process, and oxidative stress were in the biological process category. Genes involved in antioxidant activity, enzyme inhibitor activity, transporter activity, and signal transducer activity were enriched in the molecular function category, whereas DEGs involved in cell part, intracellular and extracellular region, and organelle were enriched in the cellular component category (**Figure 5D**). DEGs involved in oxidation–reduction, metabolic process, hydrogen peroxide catabolic process, catalytic activity, antioxidant activity, cell part, and apoplast were among the top 10 GO terms in the CK vs. FR_6.4_ comparison (group (**Supplementary Figure S2A**).

The functions of DEGs in the roots treated with different light conditions for 4 d were elucidated using the Kyoto Encyclopedia of Genes and Genomes (KEGG) database. KEGG analysis revealed that DEGs in the roots were primarily enriched in “starch and sugar metabolism,” “plant hormone signal transduction,” “ascorbate and aldarate metabolism,” and “hydrogen peroxide catabolic process” in the CK vs. FR_0.3_ comparison (**Figure 5E**). “Sucrose metabolism,” “plant hormone signal transduction,” “transduction’, ‘Glycolysis/Gluconeogenesis’ and ‘Galactose metabolism’ were the main pathways enriched in FR_6.4_ vs. FR_0.3_ comparison group (**Figure 5F**). In the CK vs. FR_6.4_ comparison, DEGs in the roots were strongly enriched in “starch and sugar metabolism,” “ascorbate and aldarate metabolism,” and “amino sugar and nucleotide sugar metabolism” pathways (**Supplementary Figure S2B**). Taken together, these data suggest that supplementary FR light is involved in the regulation of antioxidants, sugar metabolism, and plant hormone signal transduction, conferring a positive influence on root development in DRC grafted watermelon seedlings.

**Figure 6:**
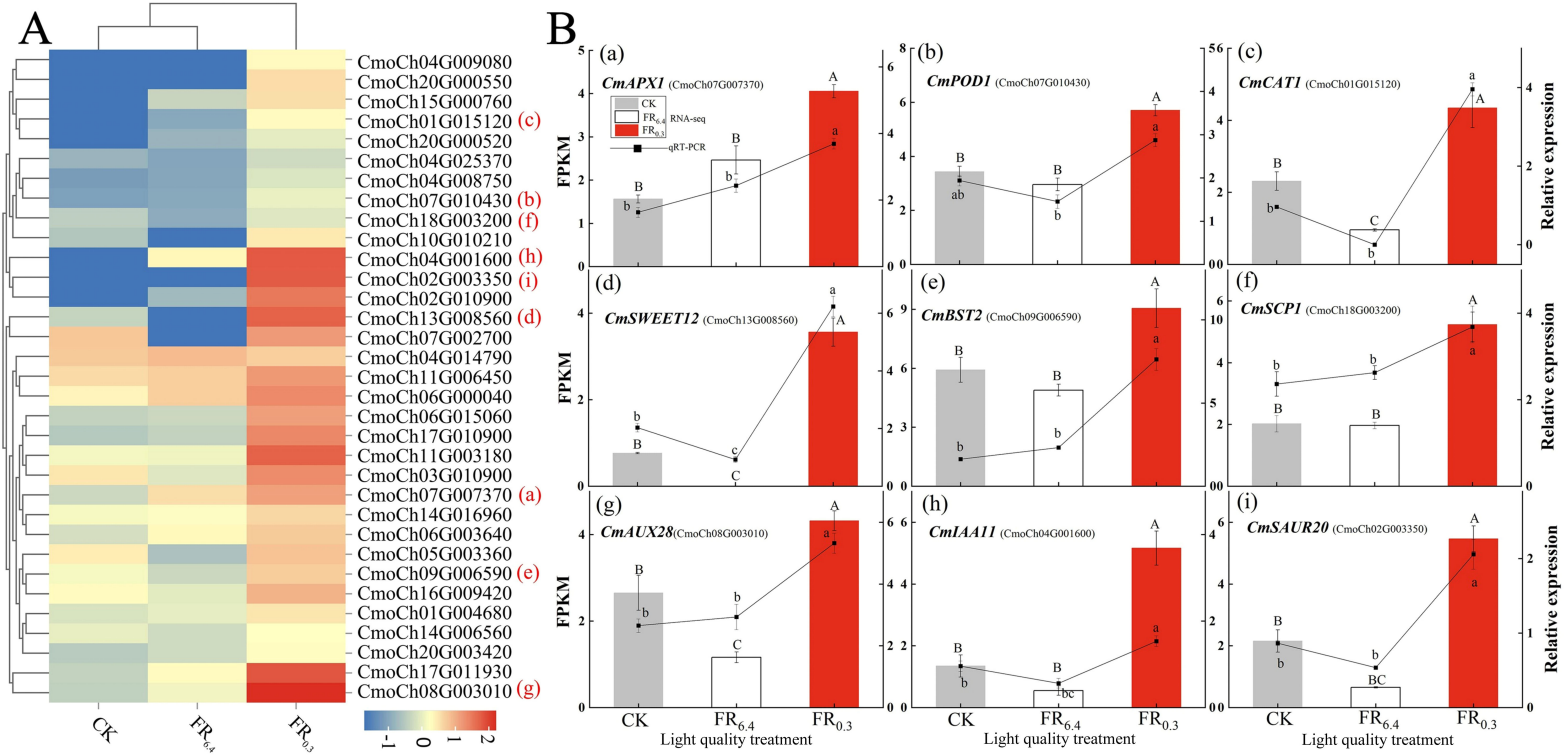
Analysis of the differently expressed genes (DEGs) associated with oxidative stress, sugar metabolism, and auxin response in the roots of DRC grafted watermelon seedlings under different light quality treatments for 4 d. **(A)** Clustering heat map of DEGs using the k-means algorithm. Y-axis: gene; x-axis: light treatments. Red: high expression; blue: low expression. **(B)** qRT-PCR validation of nine DEGs identified by RNA-seq. Statistical significance was determined by Duncan’s test (*P* < 0.05). Capital letters above bars indicate significant differences among treatments for RNA-seq results; lowercase letters indicate significant differences for qRT-PCR results. Error bars: SD of biological replications (n = 6). CK: dark control; FR_6.4_: white light; FR_0.3_: R/FR = 0.3.

Furthermore, clustering heatmap analysis was conducted to determine the transcriptional levels of DEGs involved in sugar metabolism, oxidative stress, and auxin signaling under different light treatments (**Figure 6**). The treatments were grouped into two clusters: samples treated with CK and FR_6.4_ clustered together and exhibited the fewest DEGs, whereas samples treated with FR_0.3_ formed a distinct cluster with the highest number of DEGs. The expression patterns of DEGs involved in sugar metabolism, oxidative stress, and auxin signaling were divided into nine distinct expression pattern groupings. FR_0.3_ treatment had the highest expression levels in all nine groupings compared with CK and FR_6.4_, except for glycosyl transferase (*CmoCh04G014790*) (**Figure 6A**). The expression levels of genes in CK and FR_6.4_ fluctuated depending on the gene. Specifically, FR_0.3_ significantly induced the expression of auxin-responsive genes (*CmoCh08G003010*, *CmoCh02G003350*), auxin transporters (*CmoCh04G001600*, *CmoCh11G003180*), and auxin signaling genes (*CmoCh17G011930*, *CmoCh02G010900*) compared with other light treatments. Moreover, expression of genes related to sugar metabolism was markedly increased by supplementary FR, including sugar transporters (*CmoCh13G008560*, *CmoCh11G006450*, *CmoCh09G006590*), sugar carrier proteins (*CmoCh07G002700*, *CmoCh18G003200*), and glucosidase (*CmoCh16G009420*). Transcriptional alterations in DEGs associated with ROS scavenging (*CmoCh07G007370*, *CmoCh05G003360*, *CmoCh06G003640*, *CmoCh20G000550*, *CmoCh01G015120*, *CmoCh20G000520*) were also markedly induced by FR_0.3_ treatment.

To validate the reliability of RNA-seq results, nine DEGs representing different expression change patterns were selected for qRT-PCR analysis (**Figure 6B**). In the qRT-PCR results, the expression patterns of these genes were consistent with the FPKM values from sequencing. FR_0.3_ treatment significantly induced the expression of all nine DEGs tested, especially *CmPOD1*, *CmAPX1*, *CmSWEET12* (sugar transporter), *CmIAA11* (auxin transporter), and *CmSAUR20* (auxin-responsive protein) in the roots of the rootstock, compared with CK or FR_6.4_ treatment. FR_6.4_ treatment yielded expression levels of DEGs similar to CK, except for *CmSWEET12*.

### 3.3 The ROS and antioxidant enzymes activities were affected by light treatments

**Figure 7:**
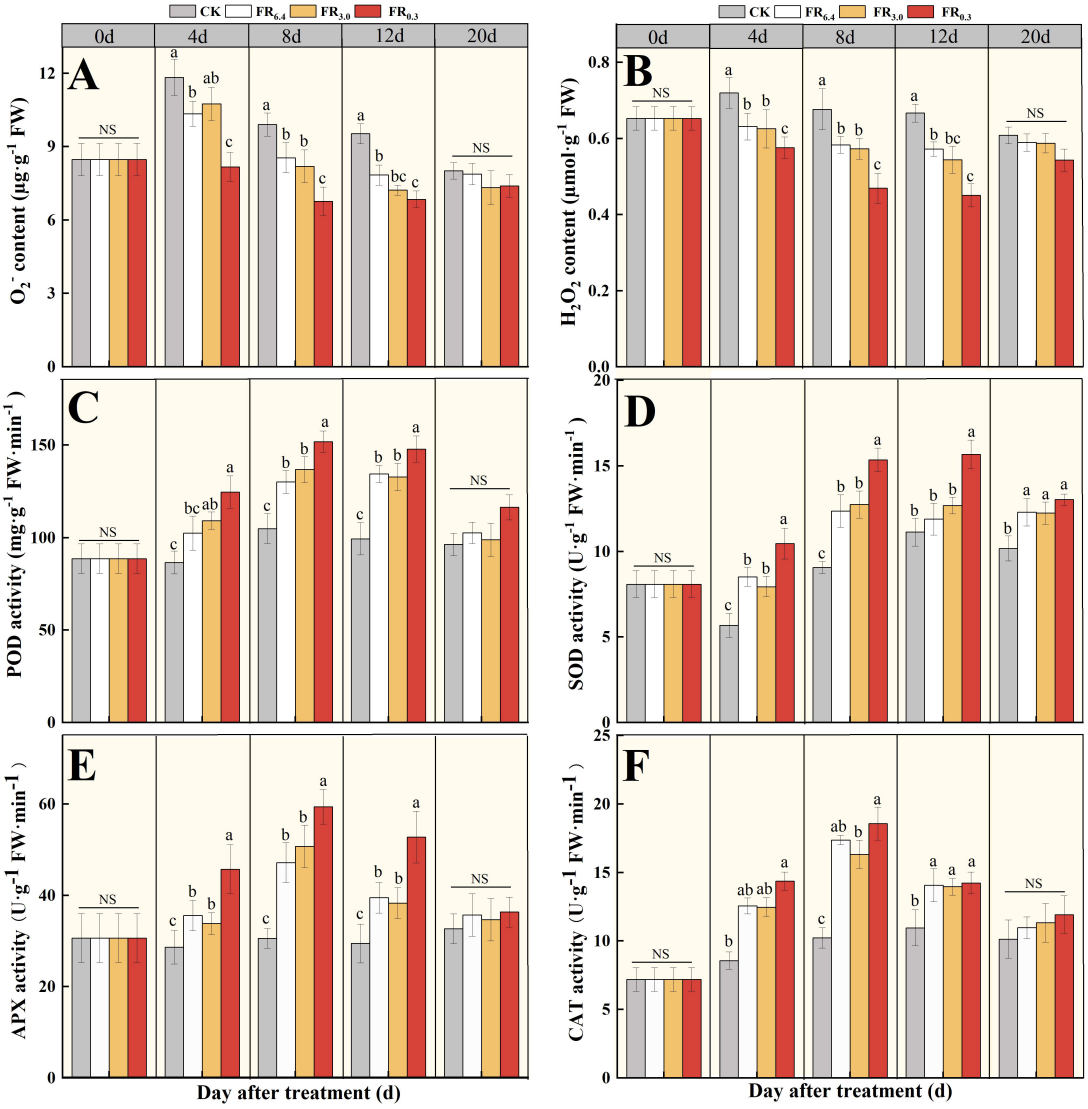
Analysis of ROS and antioxidant activities in the root of double-root-cutting (DRC) grafted watermelon seedlings under different light quality treatments for 0, 4, 8, 12, and 20 d. **(A, B)** represented the O_2_^-^ and H_2_O_2_ content, respectively; **(C–F)** represented the POD, SOD, APX and CAT activities, respectively. Statistical significance was determined by Duncan’s test (*P* < 0.05). Data are shown as means ± standard deviation (SD) from three independent experiments with three biological replicates each (n = 9). Different letters indicate significant differences at *P* < 0.05 according to Duncan’s multiple range test and the significant differences were compared among light treatments at each time point. CK: dark control; FR_6.4_: white light; FR_3.0_: R/FR = 3.0; FR_0.3_: R/FR = 0.3.

The transcriptome data and qRT-PCR results demonstrated that the transcriptional levels of hydrogen peroxide– and antioxidant-related DEGs were significantly changed after supplementary FR treatment. Therefore, O_2_^−^ and H_2_O_2_ contents, as well as POD, SOD, APX, and CAT activities in the roots of the rootstock, were measured at various days after grafting (**Figure 7**). The O_2_^-^ content first increased at 4 d after grafting for CK, FR_6.4_, and FR_3.0_ treatments, then gradually decreased over time (**Figure 7A**), whereas H_2_O_2_ concentration decreased immediately after grafting (**Figure 7B**). Roots treated with FR_0.3_ had the lowest O_2_^-^ and H_2_O_2_ contents compared with other treatments. No significant differences among treatments were observed in ROS content at 20 d after grafting.

Antioxidant enzymes can scavenge the overproduction of ROS. As shown in **Figures 6C–F**, antioxidant enzyme activities in the roots initially increased and peaked at 8 d after grafting, then decreased in all treatments. Comparatively, the light treatments showed significantly higher antioxidant enzyme activities than CK. FR_0.3_ had the highest POD, SOD, APX, and CAT activities, though not always significantly higher than other light treatments. FR_3.0_ exhibited comparable enzyme activities to FR_6.4_ at all measurement points. At 20 d after grafting, all seedlings were exposed to white light and showed similar enzyme activities, possibly because they had acclimatized to the light environment. Notably, although the control seedlings presented the lowest CAT activity, no significant differences were recorded among roots treated under FR_6.4_, FR_3.0_, or FR_0.3_ light.

### 3.4 Sugar synthesis was promoted by light treatments

**Figure 8:**
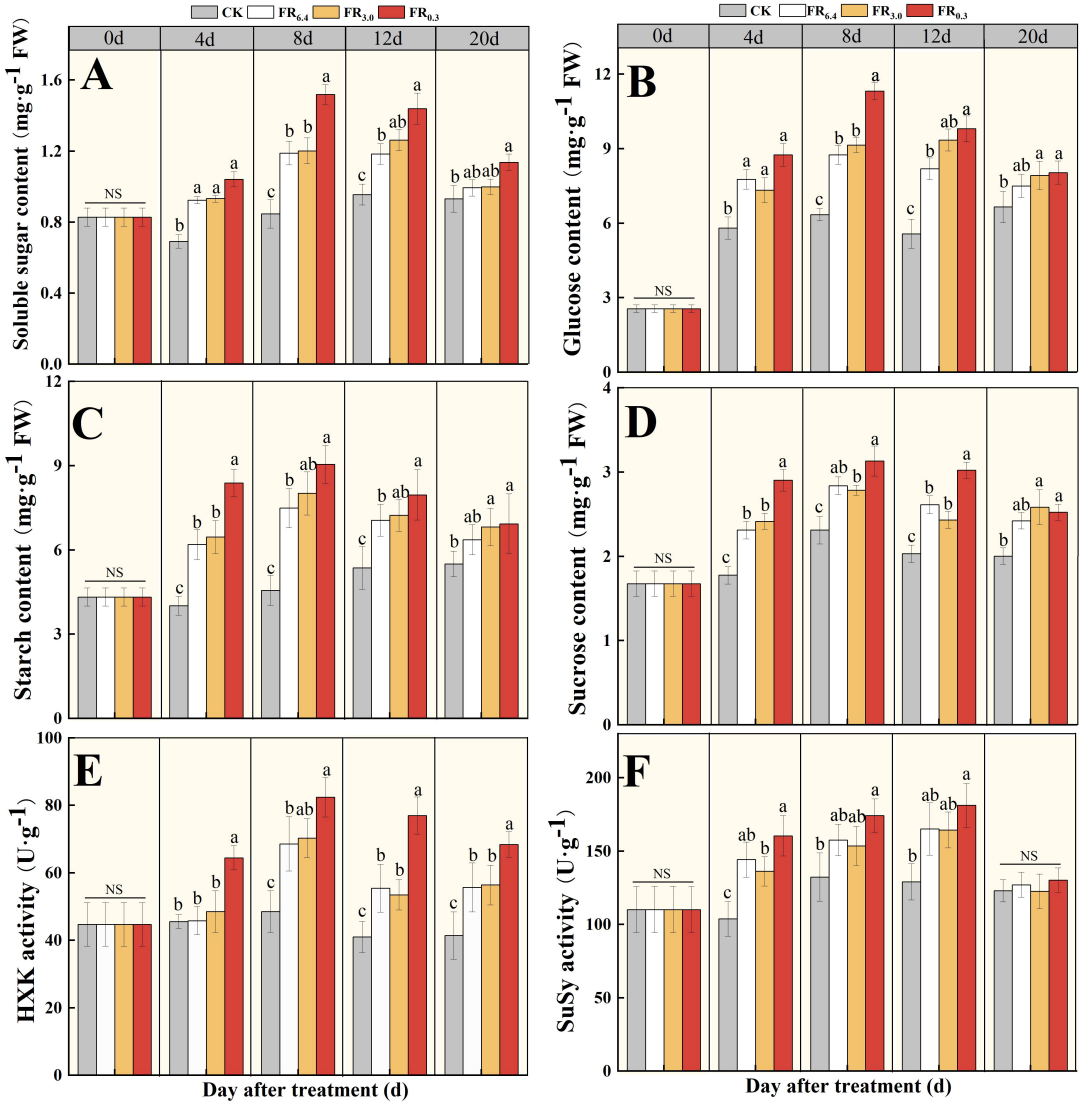
Analysis of sugar and sugar metabolism enzymes in the root of double-root-cutting (DRC) grafted watermelon seedlings with different light quality treatments for 0, 4, 8, 12 and 20 d. **(A–D)** represented the soluble sugar, glucose, starch and sucrose content, respectively; **(E, F)** represented the hexokinase (HXK) and sucrose synthase (SuSy) activities, respectively. Data are shown as means ± standard deviation SD from three independent experiments with three biological replicates each (n = 9). Different letters indicate significant differences at *P* < 0.05 according to Duncan’s multiple range test, with comparisons among light treatments at each time point. CK: dark control; FR_6.4_: white light; FR_3.0_: R/FR = 3.0; FR_0.3_: R/FR = 0.3.

The transcriptional levels of sugar-related DEGs were greatly altered by FR. Therefore, different forms of sugar (soluble sugar, glucose, starch, and sucrose) and sugar metabolism enzymes (HXK and SuSy) in the roots were further detected. As shown in **Figure 8**, the roots of seedlings under light treatments had significantly higher concentrations of soluble carbohydrates compared with the control at 4, 8, 12, and 20 d after grafting. FR_0.3_ produced the highest concentrations, although the differences from other light treatments were not always significant. Sugar content peaked at 8 d after grafting. As plants grew, the differences in sugar content among the light treatments became smaller, and no significant differences were observed between supplementary FR light and white light treatments at 20 d after grafting.

To be specific, the effects of FR_0.3_ treatment on soluble sugar content (**Figure 8A**) and glucose content (**Figure 8B**) in roots were similar. FR_0.3_ treatment showed no significant difference from other light treatments at 4 d post-grafting, but soluble sugar and glucose contents in FR_0.3_ increased greatly at 8 d post-grafting, reaching the highest levels. At 12 d post-grafting, soluble sugar and glucose contents in FR_0.3_ decreased and showed no significant difference from FR_3.0_. Starch and sucrose contents responded more rapidly to additional FR treatment, with FR_0.3_ presenting the highest contents at 4 d post-grafting (**Figures 8C, D**). Afterwards, differences among light treatments were statistically non-significant.

HXK and SuSy activities were also analyzed. FR_0.3_ had significantly higher HXK activity at 4 d and 12 d after grafting compared with other treatments (**Figure 8E**). In addition, SuSy activity was greatly promoted by light treatments compared with CK, but no significant differences were observed among the light treatments (**Figure 8F**).

## 4 Discussion

Vegetable grafting is increasingly recognized as an effective and sustainable plant production alternative (Sallaku et al., 2022). It has been reported that more than 90% of watermelon seedlings are grafted in Japan and Korea (Lee et al., 2010), and over 40% of watermelon plants are grafted in China (Zhong et al., 2018; Xu et al., 2022), of which 80% are grafted in protected production systems (Sun et al., 2023). The demand for high-quality grafted seedlings during specific seasons is high. Therefore, reducing production time is important for increasing production capacity and lowering operational costs.

Our previous research showed that additional FR light enhanced root formation in DRC grafted watermelon seedlings by regulating the transcriptional levels of PIFs and auxin-related genes through shade or FR-induced responses (Wu et al., 2024; Wang et al., 2024). Moreover, the survival rate was significantly increased by light treatments compared with CK (dark), with FR_0.3_ producing the highest survival rate (Wu et al., 2024). After multiple experimental repetitions, statistically significant differences in survival rates among different light treatments were observed (Wang et al., 2024).

The present study found that supplementary FR light significantly advanced the root formation in DRC grafted watermelon seedlings and improved seedling quality by increasing biomass and stem diameter, providing a potentially effective approach for commercial nurseries. Furthermore, the relationship between root development, ROS scavenging, and sugar accumulation was investigated. The data suggest that root regeneration was promoted by FR light and may be associated with the *PIF*-regulated synergistic effects of hydrogen peroxide catabolism, sugar metabolism, and auxin-related pathways.

### 4.1 FR light enhanced the root regeneration of DRC grafted watermelon seedlings

FR light has been reported to mediate plant growth and developmental processes, accelerate flowering, regulate plant nutrition, and shape plant morphology (Park and Runkle, 2017; Demotes-Mainard et al., 2016). A low R/FR ratio is perceived by plants as a shade avoidance response, which reduces the levels of the active form of phytochrome B and other stable phytochromes, leading to the appearance of shade avoidance syndrome (SAS) (Keller et al., 2011). SAS typically increases petiole and stem length, changes leaf angle by promoting the elongation of cells on the adaxial side, increases the leaf area intercepting light, and enhances canopy gross photosynthetic rate and biomass (Ballaré and Pierik, 2017; Ballaré, 2017; Zhen and Bugbee, 2020; Legendre and van Iersel, 2021). However, root responses to FR light have been less intensively studied.

In the last century, few investigations have addressed how the effects of R/FR ratio on plant development may influence rooting ability. Newton et al. (1996) reported a higher rooting percentage in cuttings from *Triplochiton scleroxylon* stockplants grown under a lower R/FR ratio (0.5) and noted that R/FR ratio had no effect on specific leaf area (SLA). By contrast, low R/FR ratio (0.9) treatment on stockplants increased SLA but decreased the rooting percentage of cuttings from *Terminalia* sp*inosa*. This difference in rooting response was suggested to be related to the contrasting effects of R/FR ratio on the leaf and stem morphology of the two species (Newton et al., 1996). Leakey and Storeton-West (1992) demonstrated that decreasing the R/FR ratio increased rooting ability as well as shoot extension in *Triplochiton scleroxylon* K. Schum, and proposed that the promotion effect might be attributable to the longer internode lengths—and therefore longer single-node cuttings—obtained under the lower R/FR treatment.

Recently, a positive effect of FR on rooting has also been reported for other species such as *Rhododendron*, *Chinese Thuja*, *Leucothoe* (Park et al., 2022), and *Chrysanthemum* (Christiaens et al., 2019). Although the causes of these morphological responses of root systems to shade or FR are not well understood, they could involve light perception by shoot cells and mobile signals traveling from shoots to roots (Ballaré and Pierik, 2017; Gundel et al., 2014). It has been suggested that FR upregulates the biosynthesis of auxin (Tao et al., 2008), which plays a crucial role in root formation (Vanneste and Friml, 2009); thus, FR may enhance rooting (Christiaens et al., 2016). Nevertheless, the response of roots to additional FR light may vary depending on habitat-specific adaptations, ontogenic stage, and complex interactions with other environmental factors (Gundel et al., 2014).

In this study, the increased scion height exhibited a typical SAS response, consistent with previous research, and improved root development was recorded in both DRC grafted watermelon seedlings and excised rootstock seedlings. We found that the FR_0.3_ treatment significantly expedited root regeneration (**Figures 2**, **4**), increased root length and root fresh/dry weight (**Supplementary Table S2** and S3), and elevated the root-to-shoot ratio compared with the control for both DRC grafted watermelon seedlings and excised rootstock seedlings. Christiaens et al. (2019) supported our findings by showing that the addition of FR to R (60R:60FR) resulted in faster rooting, higher root dry mass, and an increased number of primary roots in *Chrysanthemum morifolium* cuttings. They emphasized that although SAS was present under a low R/FR ratio, the positive effect of FR light on rooting predominated, as rooting was hastened and root dry mass increased. Faster root development and growth can lower costs and benefit the nursery industry.

As the additional FR light treatments were applied only for 10 days—different from natural shade conditions—and the light intensity during the first three days post-grafting was relatively low (50 μmol·m^-2^·s^-1^), the FR-induced shade avoidance in DRC grafted watermelon seedlings might have been optimized, resulting in improved growth in both above- and below-ground parts. This was further confirmed by the positive effect of FR on the growth of excised rootstock seedlings (**Figure 4**; **Supplementary Table S3**).

Nevertheless, this research had limitations. We failed to investigate the long-term effects of FR light on DRC grafted watermelon plants, such as field performance, pest and disease resistance, yield, or even fruit quality. Bantis et al. (2022b) recommended adding 5% FR to 88/12% red/blue as a light recipe for grafted watermelon seedlings, which enhanced overall growth, flowering, and yield earliness without affecting fruit quality. However, the whole growth cycle of watermelon is a much more complex process, and many factors could influence long-term assessment results. Therefore, it remains unclear whether FR light treatment during the seedling stage would have negative effects on pest and disease resistance or yield. In future experiments, DRC grafted watermelon seedlings will be planted in plastic tunnels under realistic conditions to better understand the mechanisms that control root behavior in nature and to assess field performance.

### 4.2 Oxidative stress was mitigated by light treatments during root development

The generation of reactive oxygen species (ROS) is a critical factor influencing physiological changes in grafted seedlings, such as variations in plant height, graft union hardness, and biomass accumulation (Li F.H. et al., 2021). Antioxidant enzymes, including superoxide dismutase (SOD), peroxidase (POD), catalase (CAT), and ascorbate peroxidase (APX), help maintain the balance of active oxygen metabolism in plants and safeguard the integrity of cellular membranes. As the first line of defense, SOD dismutates the superoxide radical (O_2_^−^) into hydrogen peroxide (H_2_O_2_) and molecular oxygen (O_2_). The resulting H_2_O_2_ is then eliminated by CAT, APX, and POD (Wei et al., 2020). These enzymes also play a pivotal role in the differentiation of xylem tissues at the grafting interface (Fernández-García et al., 2004).

In this study, transcriptomic sequencing results suggested that DEGs involved in the oxidation-reduction process, hydrogen peroxide catabolism, response to oxidative stress, and antioxidant activity ranked among the top five GO terms for both the CK vs. FR_0.3_ and FR_6.4_ vs. FR_0.3_ comparison groups (**Figures 5**, **6**). We further examined ROS levels, antioxidant enzyme activities, and the expression of related genes. The O_2_^−^ content increased initially, peaked at 4 days post-grafting, and then decreased (**Figure 7**), suggesting that seedlings were experiencing cutting-induced stress. In comparison, grafted seedlings under FR_0.3_ maintained a relatively stable and consistently lowest ROS level throughout the treatment period, whereas ROS levels in the control (dark) group remained elevated until 20 days post-grafting (**Figure 7**).

Additionally, the promotive effect of FR_0.3_ on antioxidant enzyme activities persisted until the end of the treatment period (12 d post-grafting). This indicates that seedlings respond rapidly to light—particularly FR light—and that exposure to FR_0.3_ facilitates the scavenging of excess ROS generated by grafting, likely through the upregulation of antioxidant enzyme activity. However, changes in CAT activity did not fully align with this hypothesis. The CAT activity in roots did not differ significantly among FR_6.4_, FR_3.0_, and FR_0.3_ treatments, although the control had the lowest CAT activity. This suggests that the increase in H_2_O_2_ under light treatments (FR_6.4_, FR_3.0_, FR_0.3_) may be insufficient to strongly activate CAT (Mittler, 2002). Therefore, the scavenging of excess ROS under FR_0.3_ treatment is likely mediated primarily by SOD, POD, and APX.

In contrast, research on *Arabidopsis thaliana* has shown that canopy shade enhances the accumulation of ROS and nitric oxide in the hypocotyl of *A. thaliana*, and favored the promotion of hypocotyl growth. This suggests that shade-induced ROS may not merely be a by-product of stress, but could play a supportive and regulatory role—particularly in modulating growth responses through hormonal and transcriptional pathways associated with the shade avoidance syndrome (Iglesias et al., 2024).

In this study, we observed the opposite ROS pattern: FR-mediated treatments decreased ROS levels while increasing plant growth in DRC grafted watermelon seedlings. This effect may be linked to modifications in phytochrome configuration induced by light treatments (Wang et al., 2021; Wu et al., 2024; Wang et al., 2024). This modification can, via the light signal transduction pathway, give rise to the accumulation of antioxidant enzyme activity and osmoregulatory substances. The more stable physiological environment created by these antioxidant systems may, in turn, benefit both root and scion growth (Xu et al., 2019).

This hypothesis was further supported by the findings of this study. The light, especially FR_0.3_ treated DRC grafted watermelon seedlings exhibited higher survival rates, root-to-shoot ratios, root fresh and dry weight, stem diameter, and root vigor (**Figures 2**-**4**, **Supplementary Tables S2**, **S3**). These improvements are likely attributable to an expanded root absorption area and enhanced plant vigor, which increase nutrient uptake capacity and stimulate overall growth. However, whether ROS indirectly contribute to the growth of DRC grafted watermelon seedlings under FR light remains uncertain. Further verification, with increased sampling frequency (especially within the first 4 days post-grafting) and systematic experimental analysis (including hormone content), will be necessary to confirm this mechanism.

### 4.3 Sugar metabolism was promoted by light treatments during root development

It is reported that carbohydrates are crucial for the regeneration of adventitious roots (Klopoteka et al., 2012). Carbohydrates are the main source of energy and provide carbon skeletons to the rooting zone to power root development and growth by supporting cell division, elongation, and differentiation into root structures (Moosavi-Nezhad et al., 2021; Haissig, 1984; Stitt and Krapp, 1999). Besides, carbohydrates also act as signaling molecules, interacting with phytohormones and regulating many developmental processes in plants, including adventitious rooting (Ruedell et al., 2015; Corrêa et al., 2005).

The adventitious root formation on grafted seedlings might be affected by carbohydrate availability, which depends on the cutting’s carbohydrate reserve. An enhanced net photosynthesis rate was noticed in the leaves of grafted seedlings after supplementary FR treatment (Wang et al., 2024) or a mixed FR + blue light treatment (Bantis et al., 2022a), which improved the seedling’s carbohydrate reserve and enhanced root regeneration. The enhanced canopy gross photosynthetic rate and biomass could be highly related to FR-induced SAS through the maximized light interception for the individual plant (Cerrudo et al., 2017; Zhen and Bugbee, 2020; Legendre and van Iersel, 2021).

Sugars produced by photosynthesis are mostly sucrose initially, which is quickly transformed into starch (Wei et al., 2020). These sugars are temporarily stored in the chloroplast and then transported to various parts of the plant. Sucrose transport plays an important regulatory role in carbon allocation and sugar signal generation (Rolland et al., 2006). Several studies have shown that phytochrome regulates sugar translocation in FR-regulated growth responses (Ranwala et al., 2002; Lercari, 1982; Yanovsky et al., 1995; Hoddinott, 1983). Ruedell et al. (2013) demonstrated that an FR-enriched environment for *Eucalyptus globulus* donor plants enhanced the rooting competence of their derived microcuttings, and suggested that this was related to the higher content of total soluble sugars and starch in the rooting zone, resulting in a high root/shoot ratio. An increased relative expression of *Sucrose Synthase 1* (*SUS1)* during the root formation period was also observed under this light treatment (Ruedell et al., 2015). It is probable that the developing roots established a new sink that competed for assimilates with the shoot meristems, and that in microcuttings from *E. globulus* donor plants exposed to FR radiation, most resources were allocated to the stem base to form new roots (Ruedell et al., 2013).

In the present study, we found that the biomass of both roots and the above-ground parts of DRC grafted watermelon seedlings was greatly enhanced by FR, especially FR_0.3_ (**Supplementary Tables S2**, **S3**). The increased carbohydrate reserve in leaves would then benefit root development. To further verify the role of sugar in FR-mediated root growth, transcriptomic sequencing was conducted. DEGs involved in sugar transport were the top GO term in the biological process category for the FR_6.4_ vs. FR_0.3_ comparison group, while sugar catabolism and sugar carrier activity were among the top five GO terms for the CK vs. FR_0.3_ comparison group (**Figure 5**). Furthermore, FR_0.3_ treatment significantly upregulated the expression of sugar transporter genes (*CmSWEET12*, *CmBST2*, and *CmSCP1*) in the roots of the rootstock (**Figure 6**).

However, the change pattern of sugar content did not fully align with gene expression (**Figure 8**). The sugar contents of FR_0.3_ treatment varied greatly during the test period. Starch and sucrose content, as well as HXK activity, responded quickly to FR_0.3_ treatment at 4 d post-grafting, whereas soluble sugar and glucose content were significantly higher only at 8 d post-grafting compared with other treatments. The initial advantage of FR_0.3_ was matched by FR_3.0_ at 8 d post-grafting (starch) or 12 d post-grafting (soluble sugar and glucose), with no significant differences observed beyond these time points (**Figure 8**). This suggests that starch synthesis and storage might be highly associated with the early stages of adventitious root formation, possibly to meet increased energy demand and metabolic activity during the root initiation phase for cell division and elongation (Ahkami et al., 2009). The difference in response time may be related to the metabolism of different sugar components and the potential involvement of other sugar metabolism gene families, as well as other types of sugars or enzymes, which could be the focus of future studies (Li et al., 2017; Ruedell et al., 2015). From another perspective, this also reflects the complexity of regulating sugar concentration in the roots of DRC grafted watermelon seedlings through additional light, making it difficult to draw a definitive conclusion on whether there is a strict positive or negative correlation between the effect and light quality (Xu et al., 2019).In addition, studies have indicated that root regeneration is associated with the rapid accumulation of auxin in the roots and leaves of grafted seedlings (Wu et al., 2024; Matosevich et al., 2020). Ruedell et al. (2015) proposed that the improved adventitious rooting of *E. globulus* microcuttings mediated by FR light treatment of donor plants was likely the result of crosstalk between auxin- and carbohydrate-metabolism-related genes and pathways, leading to enhanced rooting. Consistent with the observed changes in sugar content, the present study recorded an upregulation in the expression of auxin-related genes under FR_0.3_ treatment (**Figure 6**). Specifically, FR_0.3_ significantly increased the expression levels of *CmIAA11*, *CmAUX28*, and *CmSAUR20* compared with CK or FR_6.4_ (**Figure 6**), corroborating our earlier findings (Wu et al., 2024; Wang et al., 2024). It has been shown that auxin biosynthesis and transport are more efficient in FR-enriched environments (Tao et al., 2008; Hornitschek et al., 2012). Perhaps FR enrichment facilitates the transport of biosynthesized auxin to the stem base, thereby modulating rooting competence (Ruedell et al., 2015; Christiaens et al., 2019).

However, strong stem elongation under a low R/FR ratio might adversely affect the future growth of DRC grafted watermelon plants. In this study, additional FR light was applied only during the first 10 days of rooting, which proved sufficient to stimulate root regeneration while limiting excessive elongation (**Figures 2** and **4**; **Supplementary Tables S2** and **S3**). Even so, the long-term effects of FR on the growth of DRC grafted watermelon seedlings warrant further investigation. Sae-Tang et al. (2024) demonstrated that adding FR only during the initial stage (7 days) of rooting improved rooting of medicinal cannabis cuttings without causing excessive stem elongation. However, they found no correlation between auxin or carbohydrate content and rooting, which may have been due to sampling position or timing. In contrast to their leaf-based measurements, the concentration of auxin in the lower stem—where root initiation occurs—would likely be affected by FR exposure, but was not measured in their study (Sae-Tang et al., 2024).

Interestingly, previous research revealed that FR reduced lateral root density in *A. thaliana* by suppressing lateral root emergence through regulation of ELONGATED HYPOCOTYL5 (HY5), a transcription factor involved in the signal transduction pathways of nearly all photoreceptors (van Gelderen et al., 2018). However, non-grafted seedlings are not suitable as controls for quantitative physiological comparison with grafted seedlings in this study, due to the perturbed vasculature at the graft junction (Marsch-Martínez et al., 2013; van Gelderen et al., 2018). Grafting requires several days for recovery, and the size of grafted seedlings can vary (van Gelderen et al., 2018). Therefore, the FR-inhibited root growth mechanism reported in *A. thaliana* cannot fully explain the effect of FR on root regeneration in the rootstock of DRC grafted watermelon seedlings (Wu et al., 2024).

We also observed that although the promoting effect of FR light on root formation was consistent in both excised rootstock seedlings and DRC grafted watermelon seedlings, its impact on above-ground growth differed. Specifically, FR_0.3_ significantly enhanced the above-ground growth of excised rootstock (pumpkin) seedlings at 8 days post-grafting (**Supplementary Table S2**), but not the scion (watermelon) in DRC grafted watermelon seedlings (**Supplementary Table S3**). The substantial shoot growth observed in excised pumpkin rootstocks at day 8, used here as a non-grafted control, is therefore not directly comparable to the grafted watermelon scion at 8 days post-grafting. The graft healing process in watermelon scions takes time, which likely delays or limits their elongation response compared to non-grafted seedlings.

Taken together, supplementary light—particularly FR—can promote root regeneration in the rootstock of DRC grafted watermelon seedlings, potentially by alleviating oxidative stress from grafting and creating a relatively stable plant environment. This effect may occur through the synergistic action of sugar metabolism, antioxidant enzyme systems, and auxin accumulation in the roots, mediated by the regulation of antioxidant, sugar metabolism, and auxin-related gene transcription.

## 5 Conclusions

In general, our findings revealed that supplementary FR light significantly expedited the root development of DRC grafted watermelon seedlings. An R/FR ratio of 0.3 enhanced the expression of *CmPOD1*, *CmCAT1*, and *CmAPX1*, thereby alleviating oxidative stress associated with grafting. The accelerated seedling growth under FR light increased carbohydrate reserves in the leaves. In addition, the upregulation of *CmSWEET12*, *CmBST2*, and *CmSCP1* might promote the translocation of sugars from leaves to roots, providing carbon skeletons to the rooting zone and supporting root development and growth.

However, despite this gene upregulation, FR0.3 did not consistently result in significantly higher sugar content in roots compared with other light treatments. Meanwhile, FR also enhanced the expression of auxin-related genes (*CmIAA11*, *CmAUX28*, and *CmSAUR20*), activating the auxin pathway and further promoting root regeneration.

The findings of this study demonstrate a practical method to enhance the quality of grafted watermelon seedlings. Nevertheless, this study did not investigate the effects of FR light on yield. Future work will involve planting DRC grafted watermelon seedlings in plastic tunnels under realistic cultivation conditions to evaluate their field performance, with those results to be reported in a subsequent paper.

## Data Availability

The datasets presented in this study can be found in online repositories. The names of the repository/repositories and accession number(s) can be found in the article/**Supplementary Material**.
