# Supplementary material for: Supplementary far-red light enhances the quality and root development of double-root-cutting grafted watermelon seedlings
Source: Front Plant Sci. 2025 Sep 3;16:1586698. doi: 10.3389/fpls.2025.1586698 (PMC12440929; doi:10.3389/fpls.2025.1586698)

**Supplementary materials**


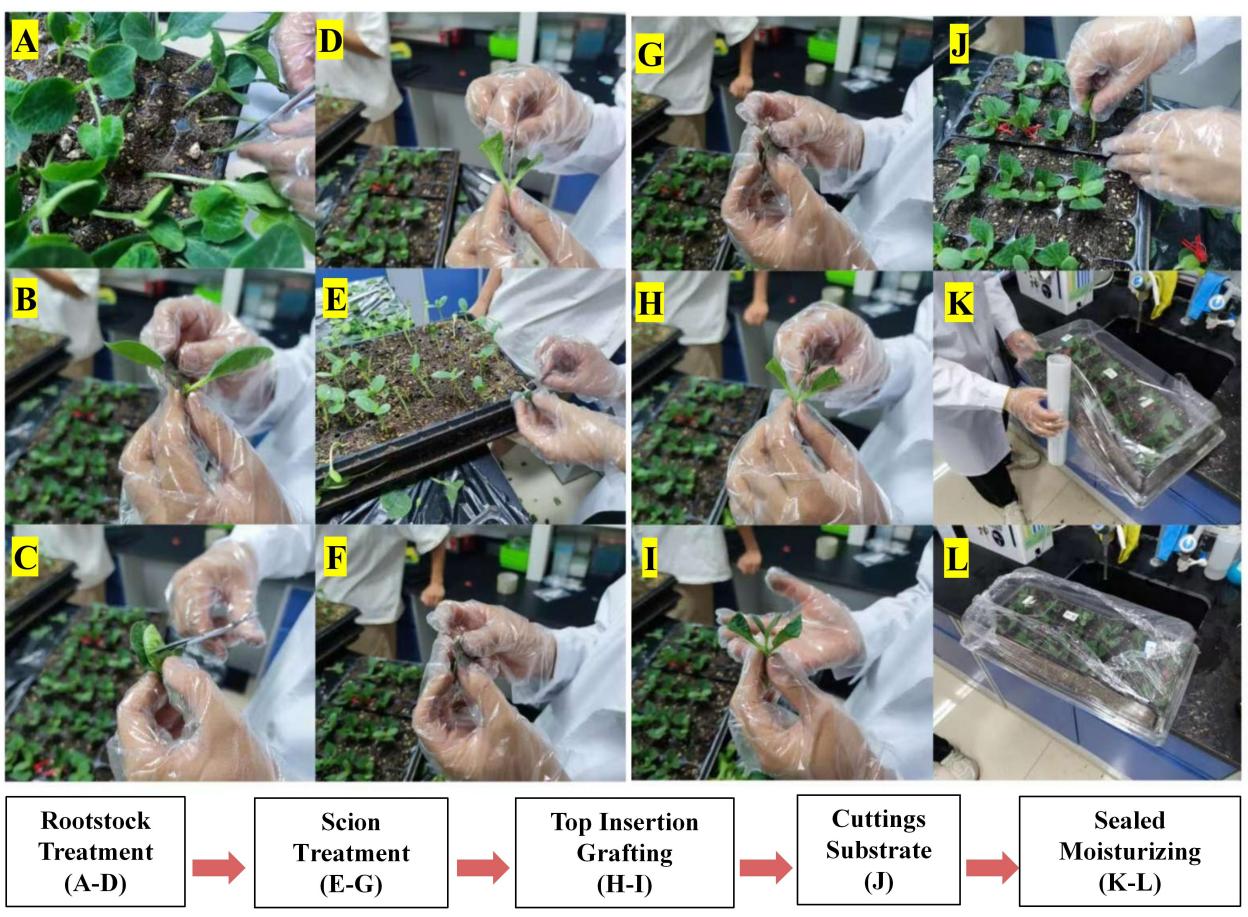


**Fig. S1.** Graphical schematic of DRC grafting**.** The rootstock with an average plant height of 13.02 ± 0.39 cm, average stem diameter of 2.40 ± 0.13 mm (n=30), was cut 5 cm below the cotyledon, then half of each rootstock cotyledon was removed to avoid mutual shading by neighbouring seedlings (A-C). A grafting needle was used to create a hole approximately 0.5 - 1.0 cm deep in the incision on the rootstock (D). A ‘V’ shaped incision was cut on scion at 1 to 2 cm below the cotyledon segment (E-G). The scion was then carefully inserted into the incision on the rootstock and held together with a plastic grafting clip (H-I). The grafted seedlings were then planted in a 72-cell plastic plug tray (54 × 28 cm) (J) and immediately covered by a matched transparent tray lid to maintain the humidity (K-L).

Table S1. Primer sequences for Real-time PCR

| **Gene name** | **Gene ID** | **Forward (5’-3’)** | **Reverse (5’-3’)** |
| --- | --- | --- | --- |
| *CmIAA11* | CmoCh04G001600 | CACTTCCTCTGCTTCTCTG | CTCTTCCTCCTCGTCTGA |
| *CmAUX28* | CmoCh08G003010 | GCTCCTTCACCACCATTG | TAGCATCCAGTCGCCATC |
| *CmSAUR20* | CmoCh02G003350 | GTACTTCTCAATGCCAAGC | CCACGTAGACTGCAATGT |
| *CmSWEET12* | CmoCh13G008560 | ACAAGAAGCAAGAGATAGCA | AGAAGGTGGTAGAGAAGAATC |
| *CmBST2* | CmoCh09G006590 | CCTACATCATCCTCTTCATAAC | AACGGAGAAGCGAACATC |
| *CmSCP1* | CmoCh18G003200 | TATAGACCTCATCTGAGTGTGG | CCACTATGGCATACCATTTGG |
| *CmCAT1* | CmoCh07G007370 | GTGGTTGGATATGGCTATGG | ACATAACAAAGCGTTGGGAGG |
| *CmPOD1* | CmoCh07G010430 | CAGAGGAAGAACTCATGGCA | GATCCAATCTGATCTTTGGTTTGG |
| *CmAPX1* | CmoCh01G015120 | TCAGCAGCGTTCATAATGC | GACAAAGTTGGCCCAGCTG |
| *CmActin* |  | CTGGACTCTGGTGATGGTGT | CGTTCAGCAGTGGTTGTGAA |

Table S2. Growth parameters of double-root-cutting grafted watermelon seedlings with different light quality treatments for 8 d and 20 d

| **Light treatment days/d** | **Parts** | **Parameters** | **Treatment** | | | |
| --- | --- | --- | --- | --- | --- | --- |
|  |  |  | **CK** | **FR_6.4_** | **FR_3.0_** | **FR_0.3_** |
| 8 |  | Plant height/cm | 10.18±0.73 b | 10.60±1.16 b | 10.32±0.65 b | 11.47±0.66 a |
|  | Scion | Stem diameter/mm | 1.45±0.08 a | 1.44±0.03 a | 1.47±0.06 a | 1.48±0.06 a |
|  |  | Fresh weight/g | 0.21±0.01 a | 0.23±0.02 a | 0.25±0.01 a | 0.32±0.01 a |
|  |  | Dry weight/g | 0.018±0.002 b | 0.020±0.001 ab | 0.020±0.001 ab | 0.022±0.001 a |
|  | Rootstock | Stem diameter/mm | 2.38±0.26 a | 2.53±0.34 a | 2.33±0.47 a | 2.62±0.76 a |
|  |  | Fresh weight/g | 1.16±0.01 a | 1.15±0.02 a | 1.15±0.04 a | 1.17±0.01 a |
|  |  | Dry weight/g | 0.048±0.003 a | 0.049±0.003 a | 0.046±0.004 a | 0.047±0.002 a |
|  | root | Root length/cm | 2.09±0.53 c | 4.06±0.68 b | 3.87±0.56 b | 4.64±0.60 a |
|  |  | Fresh weight/g | 0.103±0.006 c | 0.144±0.012 ab | 0.139±0.014 b | 0.158±0.003 a |
|  |  | Dry weight/g | 0.003±0.0003 b | 0.003±0.0002 b | 0.004±0.0001 b | 0.005±0.0004 a |
| 20 |  | Plant height/cm | 25.83±1.75 c | 32.41±4.57 b | 27.30±5.28 c | 43.27±5.56 a |
|  | Scion | Stem diameter/mm | 2.38±0.47 b | 3.31±0.33 a | 3.34±0.25 a | 3.47±0.49 a |
|  |  | Fresh weight/g | 1.03±0.37 c | 3.49±0.26 b | 3.06±0.76 b | 4.98±0.91 a |
|  |  | Dry weight/g | 0.099±0.036 c | 0.249±0.054 b | 0.249±0.067 b | 0.371±0.064 a |
|  | Rootstock | Stem diameter/mm | 4.27±0.47 b | 4.58±0.47 b | 4.51±0.58 b | 5.50±0.72 a |
|  |  | Fresh weight/g | 2.24±0.26 a | 2.48±0.37 a | 2.28±0.31 a | 2.52±0.31 a |
|  |  | Dry weight/g | 0.151±0.030 a | 0.159±0.032 a | 0.140±0.021 a | 0.165±0.024 a |
|  | root | Root length/cm | 14.31±1.49 c | 17.23±3.36 ab | 15.63±2.60 b | 19.43±3.60 a |
|  |  | Fresh weight/g | 0.65±0.21 c | 1.09±0.21 b | 0.94±0.25 b | 1.35±0.38 a |
|  |  | Dry weight/g | 0.028±0.010 c | 0.049±0.008 b | 0.045±0.010 b | 0.064±0.012 a |

Note: Within the same column, different letters represent significant differences among treatments (*P*<0.05). CK: dark as control, FR_6.4_: white light, FR_3.0_: R/FR ratio=3.0, FR_0.3_: R/FR ratio =0.3.

Table S3 Growth parameters of excised rootstock (pumpkin) seedlings with different light quality treatments for 8 d and 20 d

| **Light treatment days/d** | **Parameters** | | **Treatment** | | | |
| --- | --- | --- | --- | --- | --- | --- |
|  |  |  | **CK** | **FR_6.4_** | **FR_3.0_** | **FR_0.3_** |
| 8 | Stem diameter/mm | | 3.32±0.05 b | 3.38±0.04 ab | 3.38±0.05 ab | 3.49±0.05 a |
|  | Above-ground | Height/cm | 8.93±0.19 b | 8.83±0.13 b | 8.90±0.15 b | 9.91±0.24 a |
|  |  | Fresh weight/g | 1.34±0.04 b | 1.47±0.03 b | 1.45±0.05 b | 1.72±0.06 a |
|  |  | Dry weight/g | 0.073±0.002 b | 0.076±0.002 b | 0.082±0.003 b | 0.095±0.004 a |
|  | Below-ground | Root length/cm | 2.94±0.20 c | 4.67±0.37 b | 6.05±0.53 b | 7.49±0.34 a |
|  |  | Fresh weight/g | 0.065±0.006 c | 0.136±0.010 b | 0.168±0.014 a | 0.191±0.008 a |
|  |  | Dry weight/g | 0.003±0.0001 c | 0.007±0.0007 b | 0.007±0.0004 b | 0.009±0.0004 a |
| 20 | Stem diameter/mm | | 3.96±0.08 a | 4.02±0.08 a | 4.06±0.10 a | 4.22±0.09 a |
|  | Above-ground | Height/cm | 21.81±0.58 c | 24.37±0.55 b | 26.48±0.72 a | 26.89±0.75 a |
|  |  | Fresh weight/g | 8.35±0.24 c | 9.97±0.45 b | 10.99±0.30 b | 12.98±0.61 a |
|  |  | Dry weight/g | 0.54±0.01 c | 0.63±0.01 b | 0.65±0.01 b | 0.81±0.02 a |
|  | Below-ground | Root length/cm | 24.50±0.73 b | 24.63±1.00 b | 24.09±0.59 b | 27.85±1.02 a |
|  |  | Fresh weight/g | 1.24±0.07 c | 1.51±0.09 bc | 1.62±0.06 b | 2.22±0.04 a |
|  |  | Dry weight/g | 0.043±0.001 c | 0.059±0.001 b | 0.061±0.001 b | 0.077±0.001 a |

Note: Within the same column, different letters represent significant differences among treatments (*P*<0.05). CK: dark as control, FR_6.4_: white light, FR_3.0_: R/FR ratio=3.0, FR_0.3_: R/FR ratio =0.3.

**Fig. S2.** Gene ontology (GO) enrichment, Kyoto Encyclopedia of Genes and Genomes (KEGG) enrichment analyses of differentially expressed genes (DEGs) in the root of double-root-cutting (DRC) grafted watermelon seedlings with different light quality treatments for 4 d. (A) Comparative GO terms between CK vs. FR_6.4_. Y-axis represented GO term, while x-axis represented number of DEGs. (B) DEGs in CK vs. FR_6.4_ comparison group. Y-axis represented pathways, while x-axis represented rich factor. The rich factor meant the degree of DEGs enrichment in each pathway, the bigger the rich factor the greater the DEGs enrichment. The qvalue was the pvalue which was corrected by multiple hypothesis testing. The closer to zero the qvalue was, the more DEGs clustered (0 < qvalue < 1). The round dots of various sizes represented the number of DEGs clustered in each pathway, the larger the dot, the more the DEGs (*P* < 0.05). The various colors of the dots correspond to the different qvalue range. CK: dark as control, FR_6.4_: white light.


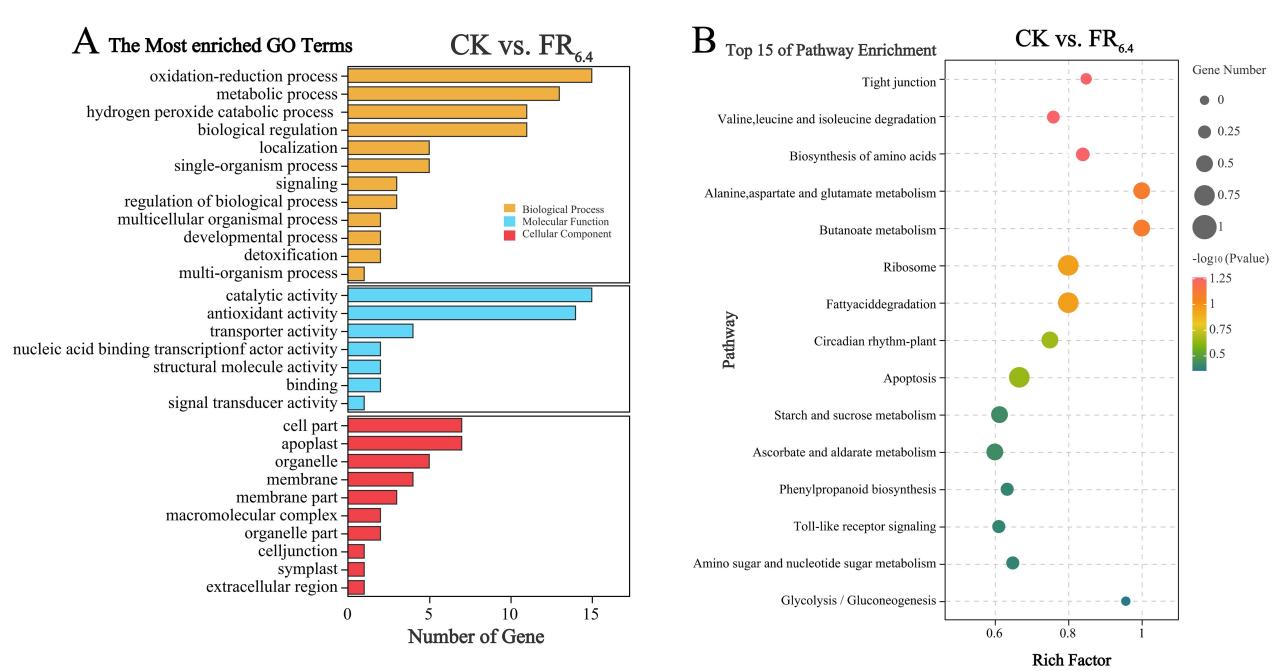

Supplement: Supplementary file 1 [file DataSheet1.docx]
